# Recruitment and Retention of Parents of Adolescents in a Text Messaging Trial (MyTeen): Secondary Analysis From a Randomized Controlled Trial

**DOI:** 10.2196/17723

**Published:** 2021-12-20

**Authors:** Joanna Ting Wai Chu, Angela Wadham, Yannan Jiang, Karolina Stasiak, Matthew Shepherd, Christopher Bullen

**Affiliations:** 1 The National Institute for Health Innovation School of Population Health University of Auckland Auckland New Zealand; 2 Department of Psychological Medicine Faculty of Medical and Health Sciences University of Auckland Auckland New Zealand; 3 School of Psychology Massey University Auckland New Zealand

**Keywords:** parenting, mHealth, text messaging, recruitment

## Abstract

**Background:**

Parenting programs are well established as an effective strategy for enhancing both parenting skills and the well-being of the child. However, recruitment for family programs in clinical and nonclinical settings remains low.

**Objective:**

This study aims to describe the recruitment and retention methods used in a text messaging program (MyTeen) trial for parents of adolescents (10-15 years) and identify key lessons learned. We aim to provide insights and direction for researchers who seek to recruit parents and build on the limited literature on recruitment and retention strategies for parenting program trials.

**Methods:**

A recruitment plan was developed, monitored, and modified as needed throughout the course of the project. Strategies to facilitate recruitment were identified (eg, program content and recruitment material, staff characteristics, and study procedures). Traditional and web-based recruitment strategies were used.

**Results:**

Over a 5-month period, 319 parents or caregivers expressed interest in our study, of which 221 agreed to participate in the study, exceeding our recruitment target of 214 participants. Attrition was low at the 1-month (4.5% overall; intervention group: n=5, 4.6%; control group: n=5, 4.5%) and 3-month follow-ups (9% overall; intervention group: n=10, 9.2%; control group: n=10, 8.9%).

**Conclusions:**

The use of web-based recruitment strategies appeared to be most effective for recruiting and retaining parents in a text-messaging program trial. However, we encountered recruitment challenges (ie, underrepresentation of ethnic minority groups and fathers) similar to those reported in the literature. Therefore, efforts to engage ethnic minorities and fathers are needed.

**Trial Registration:**

Australian New Zealand Clinical Trials Registry ACTRN12618000117213; https://www.anzctr.org.au/Trial/Registration/TrialReview.aspx?id=374307

## Introduction

Parenting programs, aimed at strengthening parenting skills and increasing knowledge on adolescent development, have shown positive effects on parent-adolescent relationships and parent-adolescent well-being [1-3]. However, recruitment for family programs in clinical and nonclinical settings remains low [4,5]. Studies have shown that only 10% to 31% of eligible parents enroll to participate in face-to-face programs—the most common mode of delivery for parenting intervention, with up to one-third of enrolled participants not attending a single session [6]. Many studies on parenting programs find it challenging to recruit an adequate number of participants for sample requirements and obtaining a representative sample of their target population [7,8]. Such challenges can result in extended recruitment time, increased costs, underpowered studies, or limited generalizability. Although an increasing number of strategies and approaches on how to boost or optimize recruitment are now known [4,6,7], the knowledge of experience from studies on parenting adolescent populations is limited.

Recently, there has been a surge of interest in the development of mobile health (mHealth) interventions as a means of expanding intervention reach [9-11]. Text messaging, in particular, has emerged as a fast and accessible mode for intervention delivery, as it minimizes many of the barriers contributing to the low uptake and attendance in traditional delivery models [9]. There is, however, limited evidence on the effectiveness of using text message as a mode of delivery for parenting programs [12]. Of those available, parenting programs have primarily targeted parents with young children [13,14]. Moreover, no study has reported on the experience with recruiting parents of adolescents into a text-messaging program trial. In 2018, we developed and trialed a text messaging program (MyTeen) with the goal to improve parenting competence and mental health literacy [2] for parents with adolescents (10-15 years of age). The 4-week-long program consisted of a series of one-way messages to participating parents that provided tips on a wide range of parenting-related matters—establishing and maintaining positive relationships with adolescents, strategies to increase adolescent autonomy, adolescent development, family functioning, parental self-care, recognizing depressive symptoms, understanding treatment options, and providing links to evidence-based support and informational resources. The text messages were derived from the Parenting Strategies Program [15], a set of evidence-based parenting guidelines developed through a systematic review and meta-analysis of parental factors associated with adolescent depression and anxiety, and international expert consensus achieved via a Delphi study about actionable strategies parents can use to reduce their child’s risk of depression and anxiety. We conducted a randomized controlled trial to evaluate the effectiveness of the MyTeen program in comparison with a “care as usual” control group [16].

In this paper, we describe the recruitment and retention methods used in the MyTeen trial. This is the first study to systematically document the process and identify key lessons learned from a text-messaging parenting program for parents of adolescents. We aim to report on our recruitment experience with MyTeen to support parents of adolescents. The paper provides insights and direction for researchers who seek to recruit parents and build on the limited literature on recruitment and retention strategies for parenting program trials.

## Methods

This section provides an overview of the study design of the MyTeen trial, including the recruitment plan developed.

### Study Design and Sample Size

The study was approved by the University of Auckland Human Participants Ethics Committee (UAHPEC, Ref 019659), and the study protocol has been published elsewhere [2]. Briefly, eligible parents or caregivers (hereafter referred to as parents) were randomly allocated to the MyTeen intervention program or care-as-usual condition. Data were obtained from all participants at baseline and at 1 month (end of intervention phase) and 3 months postrandomization. The trial is registered with the Australian New Zealand Clinical Trials Registry (ACTRN12618000117213).

We aimed to recruit a representative sample of 214 parents (n=107 per randomized group; 1 parent per household) residing in New Zealand across a 6-month period. This sample size provided 80% power (*P*=.05) to detect a group difference of 2.5 (SD 5.8) in the primary outcome measure of Parenting Sense of Competence scale (PSOC) score at the 1-month follow-up and allowing for an estimated 20% loss to follow-up. The majority of the New Zealand population is of European descent (70%), followed by indigenous Māori (16.5%), Asian (15.3%), and Pacific (9%) descent [17]. Effort was made to oversample ethnic subgroups in order to allow for subgroup analyses. Parents were eligible for inclusion in the study if they (1) had a child aged between 10 and 15 years, (2) had access to a mobile phone, (3) were not receiving any professional assistance for their own and/or child’s mental health problems, (4) possessed adequate knowledge of the English language, and (5) provided informed consent. Only 1 parent from each household was recruited for the study. Parents who showed high level of stress, as reported by the Parental Stress Index (ie, score ≥72), were excluded from the study and directed to professional services. Interested individuals completed a phone screening to assess eligibility criteria and provide contact information. Eligible individuals were sent an email through which they provided informed consent and completed a baseline survey.

### Recruitment Plan

Strategies for successful recruitment and retention were considered at the onset of the project, and a recruitment plan was developed, monitored, and modified as needed throughout the project. Potential barriers (eg, budget constraints, timeframe, and attrition) and strategies to facilitate recruitment (eg, program content and recruitment material, staff characteristics, recruitment strategies, and study procedures) were identified. Each of these strategies are outlined below.

#### Program Content and Recruitment Material

A key factor to program success was to ensure that the program met the needs of the targeted population. To this end, formative work was conducted comprising 5 focus groups (n=45) of parents or primary caregivers of adolescents (10-15 years) to ensure the content, duration, and mode of delivery were acceptable and feasible for these parents. We examined the parents’ perspectives on youth well-being, parenting, and parenting support and their input on the development of MyTeen text messaging parenting program (details reported elsewhere [18]). We found that participants were concerned about their child’s mental health, and a number of parenting challenges (ie, social expectations, time, impact of technology, changes in family communication pattern, and recognizing and talking about mental health issues) were noted. Importantly, participants reported the lack of services and support available for families, and many were not aware of services for parents themselves. Parents offered suggestions for the MyTeen program, including the tonality, content, and length of text messages, as well as their delivery frequency. These suggestions helped fine-tune the program with positively framed text messages that provided parents with strength-based parenting strategies, wordings of encouragement, and support. This also guided the wording and design of the recruitment material (eg, flyers and Facebook ads), including the use of positive and lay language (Multimedia Appendix 1). The intent was to normalize and reduce stigma to access parenting support and, in this case, the study trial. Contact details were obtained from focus group participants who expressed interest in being part of the text messaging program trial.

#### Research Staff Characteristics

One project manager and 2 research assistants conducted the recruitment, retention contacts, and logistical arrangements, with oversight by the principal investigator (JC). One of the research assistants who identified as Māori (indigenous people of New Zealand) actively engaged with ethnic minorities via her own networks, as well as promoted visibility of the program within the Māori community. Primary recruitment activities included communicating with various organizations and networks, reviewing enrolment reports, communicating the enrolment status to the steering committee, and monitoring social media and communications with our data management team.

#### Recruitment Strategies

##### Overview

The proposed recruitment period was 6 months. However, we reached our targeted sample within 5 months (March 2018 to August 2018). Table 1 details the recruitment strategies used over time. Recruitment strategies included a mix of traditional (eg, information provided to schools, distribution of flyers, word of mouth) and web-based (eg, advertising on websites, direct emails, and social media) methods. Each method was monitored on an ongoing basis and modified as needed based on recruitment success. All sources of recruitment directed interested individuals to contact us via email or phone managed by our research assistants.

**Table 1:** Recruitment strategies used over time^a^.

Recruitment strategies	Week																														
	0	2		4			6		8		10		12		14		16		18		20		22		24		26		28		
Targeted minority recruitment	✓		✓		✓																										
Flyers	✓		✓		✓																										
Community event				✓							✓																				
Social media (eg, Facebook)				✓					✓				✓																		
Paid Facebook ad																			✓												
Email Listserv							✓																								
School newsletter											✓						✓				✓		✓								
Website advertisement													✓																		

^a^Word of mouth is not shown in the table as it was used throughout the recruitment period.

Specifically, recruitment strategies varied by site or context, as described below.

##### Schools

Emails explaining the study process and asking for permission to advertise via schools were sent to 388 schools across New Zealand. Of those, 7 (1.8%) schools included our advertisement in their e-newsletter.

##### Flyers

Approximately 50 hard copies of flyers were distributed in the community via community events and local and community organizations. Community organizations and individuals were encouraged to forward or share the information among others who might be interested. The visibility of the flyers in the community helped provided legitimacy and familiarity of the study and made initial contacts more positive.

##### Word of Mouth

Participants were also recruited via word of mouth, with the message spread among local community organizations. Participants who enrolled in the study were also encouraged to share and inform others who might be interested, serving as agents to expand recruitment.

##### Advertising on Websites

A free editorial piece was written for a website that provided information, guides, and events in Auckland for families with children. The website was widely accessed by parents, with over 37,000 followers on their Facebook page. The study was also advertised on the University of Auckland’s research opportunity website.

##### Email Listserv

A recruitment email describing the study was sent to demographically diverse email lists of organizations across New Zealand, including the University of Auckland and “Health Promoting School” (now inactive), with subscribers comprising educators and health professionals. Individuals were encouraged to forward the recruitment email to parents who might be interested.

##### Social Media

A number of community organizations were approached via email and personal network for permission to post our advertisement on their social media pages. Of the 47 organizations approached, 9 (19.1%) promoted our study and posted the advertisement on their social media pages. Furthermore, a paid Facebook ad post was set up during week 18 of recruitment, and it lasted for 2 weeks and targeted parents who resided in New Zealand. We monitored the performance of the ad campaign, as response drop-offs were common over time.

##### Targeted Minority Recruitment

Multiple strategies were used to recruit the ethnic minority. These included focused outreach efforts utilizing social networks of our research team and emphasized heavily on direct person-to-person contacts and community referrals. In addition to initiating contacts with key members of the community, our research staff also relied on other events and group settings that involved the target community, such as community events, church groups, and sports clubs, where they informally provided information about the study.

### Study Procedures

Care was taken to minimize participant burden, a factor that likely contributes to study enrolment and retention [19], to engage participants throughout the trial, and to maximize retention. Specifically, we anticipated that the delivery of the program via text messages would be a possible way to minimize participant burden by reducing logistic barriers for parents. Efforts were made to ensure that data collection at each time point was brief and took no longer than 10 minutes for participants to complete. Overall, each participant needed to spend only 1 hour (including providing study information, screening, baseline, and 1- and 3-month follow-ups), across a 3-month period, to complete the study, over and above the time to receive the program.

Screening and eligibility of interested participants were assessed over the phone. Our research assistant provided information about the study and made sure that the participants understood the importance of follow-up data collection being essential and integral to the research. Participants were explicitly told that participation involves completion of 3 sets of questionnaires at various time points. Eligible individuals had 2 weeks to provide consent and complete the baseline assessment. Personalized reminder emails were sent to eligible individuals between 3 and 5 days postscreening if they had not completed the assessment. On day 10, a phone call was made to remind the study participants to complete the baseline assessment. Up to 3 emails and 2 phone calls were made before the eligible participant was deemed unable to contact or as someone who refused participation.

Assessments were conducted immediately post intervention (1 month) and 3 months after randomization. To maximize data retention at each assessment, multiple methods of communications were used to support participant retention, including texting, emailing, and phone calls. Five days before the assessment was due, participants were sent a reminder email to thank them for their participation and remind them about the upcoming assessment. For the control group, the email also specified that the participant would have the option to receive MyTeen text messages upon completion of the final assessment. For participants who did not complete the assessment within 3 days of the assessment email, up to 2 text message reminders (3-4 days apart) were sent and a final email or phone call was sent after 2 weeks of noncompletion. We incentivized participants with a NZ $20 (US $13.60) supermarket voucher upon completion of all assessments and the option to be included in a draw for a supermarket voucher valued at NZ $150 (US $102).

## Results

### Recruitment Tracking

**Figure 1:**
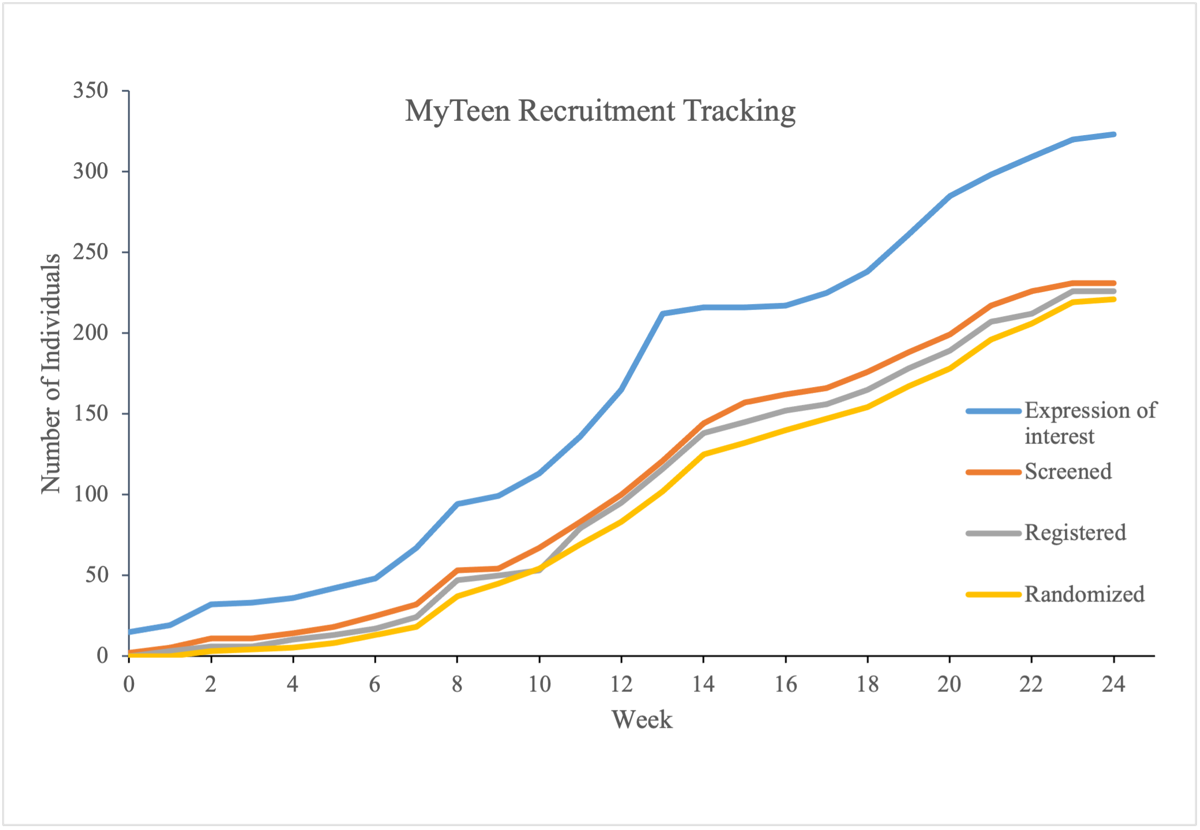
Number of participants who expressed interest in the study over time.

Figure 1 shows the number of participants who expressed interest over time. We were unable to quantify successful enrolment for each strategy separately as they were not independent. Recruitment was tracked by the project manager (AW) and reported to the research team weekly. For the first 4 weeks, most of the recruitment effort focused on targeted minority recruitment. However, recruitment was slow, and only 22 individuals expressed interest, excluding those who expressed interest from the focus groups conducted during the development stage of the project (n=15). The research team therefore targeted the wider community and distributed advertising material through email lists and social media over the next 6 weeks, resulting in a surge in interest (n=93). By week 12, a total of 200 individuals had expressed interest, and our research assistants were at full capacity to screen all potential participants. Decision was therefore made to put recruitment on hold and resumed in week 16. After reviewing the demographic profile of all participants, a paid Facebook ad targeting ethnic minority groups was posted. A number of schools were also contacted for recruitment to increase the chance of recruiting minority groups. Over 22 weeks, 319 parents expressed interest in the study, at which point, all recruitment activities were ceased. Screening was conducted over the phone with all interested individuals; 50 (15.7%) participants were no longer contactable, 18 (5.6%) participants were no longer interested, and 15 (4.7%) participants were deemed ineligible prior to completing the screening process. In total, 236 (74%) participants completed screening, of which 48 (20.3%) reported hearing about the study via email; 64 (27.1%), via advertisements (websites); 64 (27.1%), via Facebook; 29 (12.3%), via referral, including word of mouth and face-to-face approaches; and 31 (13.1%), via other means (eg, schools). No specific strategy appeared to be more engaging for Māori and Pacific participants, which is likely due to the small sample of ethnic minorities. Similarly, due to the small sample of fathers, no difference was observed among different recruitment strategies. Data on demographics were obtained at baseline.

### Sample Characteristics

Table 2 presents the demographic characteristics of the study sample. The final sample resulted in 221 randomized participants who met the eligibility criteria, exceeding our recruitment target of 214 participants. The sample comprised 210 (95%) mothers (including stepmothers), with a majority (167/221, 75.6%) of participants identifying themselves as European, followed by Māori (29/221, 13.1%), Pacific (17/221, 7.7%) and other (8/221, 3.6%).

**Table 2:** Demographic characteristics of the study sample classified by ethnicities.

Characteristic			Maori (n=29)		Pacific (n=17)			Non-Maori, non-Pacific (n=175)			
Child’s age (years), mean (SD)			12.4 (1.5)		12.2 (1.7)			12.3 (1.6)			
**Child’s sex, n (%)**											
	Female	14 (48.3)		7 (41.2)			79 (45.1)				
	Male	15 (51.7)		10 (58.8)			96 (54.9)				
**Relationship to the child, n (%)**											
	Mother	27 (93.1)		15 (88.2)			166 (94.9)				
	Father	1 (3.4)		1 (5.9)			5 (2.9)				
	Stepparent	0 (0)		1 (5.9)			1 (0.6)				
	Grandparent	1 (3.4)		0 (0)			2 (1.1)				
	Close relative	0 (0)		0 (0)			1 (0.6)				
**Marital status, n (%)**											
	Married or de facto	20 (69)		14 (82.4)			183 (82.8)				
	Divorced, separated, or widowed	7 (24.1)		2 (11.8)			30 (13.6)				
	Never married	2 (6.9)		1 (5.9)			8 (3.6)				
**Education level, n (%)**											
	University	13 (44.8)		11 (64.7)			144 (82.3)				
	Trade or technical college	4 (13.8)		0 (0.0)			6 (3.4)				
	High school or less	7 (24.1)		5 (29.4)			19 (10.9)				
	Other	5 (17.2)		1 (5.9)			6 (3.4)				
**Family structure, n (%)**											
	Original family	17 (58.6)		12 (70.6)			130 (74.3)				
	Stepfamily	3 (10.3)		2 (11.8)			15 (8.6)				
	Sole parent family	6 (20.7)		2 (11.8)			22 (12.6)				
	Living with extended family	3 (10.3)		0 (0)			9 (3.4)				
	Other	0 (0)		1 (5.9)			2 (1.1)				

### Attrition

Attrition was low at the 1-month (4.5% overall; intervention group: n=5, 4.6%; control group: n=5, 4.5%) and 3-month (9% overall; intervention group: n=10, 9.2%; control group: n=10, 8.9%) follow-ups. On average, participants in the intervention and control groups took 3.72 (SD 5.43) and 2.33 (SD 3.83) days, respectively, to complete the 1-month assessment, and 3.82 (SD 6.74) and 4.09 (SD 7.71) days, respectively, to complete the 3-month assessment.

## Discussion

Our recruitment efforts were successful—the target sample size was achieved, with a high completion rate for the trial and within the anticipated time frame. However, we did not achieve the representative demographic makeup (ethnicity and socioeconomic variables) in our trial. Below, we describe and reflect on the lesson learned.

### Traditional Recruitment Strategies

First, reliance on traditional recruitment methods, such as distribution of flyers and posters, targeted minority recruitment via word of mouth, and community referral was not particularly effective. There were few referrals from community organizations where we had posted our flyers. Similarly, handing out flyers at community events resulted in limited responses. It is likely that merely posting and handing out these flyers was not enough in these settings. Recruitment of parents from schools attended by their children was also not very fruitful. In all, 388 schools nationwide were contacted for recruitment, but only a few responded. Nonetheless, those that did advertise our study spiked an increase in expression of interest. Our findings on engagement with schools are similar to that of other studies [20]. A previous study that recruited parents of primary school students into a smoking cessation trial reported similar challenges, wherein only 16.3% of the schools contacted agreed to distribute recruitment materials [20]. Although schools can be a valuable resource for recruitment, gaining access to schools proved to be very challenging and time consuming. Studies that have successfully worked with schools to recruit participants (usually students) suggest that it requires extensive planning (ie, fitting the timeframe of the school terms), building relationship with the school, connecting with key contacts, and contributing to the best interest of schools [21]. This may not be feasible for studies that are resource constrained.

### Web-Based Strategies

Web-based strategies appear to have yielded the most response in our trial. A number of parents responded to our Facebook post and website advertisements. The use of email listserv also appeared to have yielded a spike in interest; however, it was not possible to know how many parents were reached, as individuals were encouraged to forward the email to others who might be interested in the study. It is worth noting that the ability of email lists to target a select population may result in a sample that is not representative of all parents. Our advertisement email sent to the University listserv resulted in sample of highly educated parents, which was thus less representative of the general population. Obtaining permission to post to listservs, which may accept posts only from group members, can also be challenging. Nonetheless, this approach required minimum staff effort. Studies examining web recruitment strategies have reported large variation in how many participants researchers can recruit, cost per participant, diversity of the sample, and the length of time required for recruitment [4,22]. This has implications for researchers and areas of study that may not have the funding required to enable large-scale recruitment using more traditional recruitment methods [4].

### Reaching Ethnic Minorities

Second, although we actively sought to recruit ethnic minorities, we failed to attract interest. We were aware of our limitations at the onset of the project in recruiting minority populations but were restricted by resources and time to address the challenges. We recognize that recruitment strategies should be culturally sensitive and tailored to the needs of a given group. Time to build relationships and resources to comprehensively reach a community is a requisite. By developing a partnership with trusted individuals and organizations early during the research process, researchers can build a bridge to communities that may feel disenfranchised from traditional academic research [7,23,24]. Although these strategies are intensive and expensive to build and sustain [24], they are essential if the experience of these groups with interventions is to be evaluated.

### Successful Recruitment Factors

In addition to the strategies used, the success of our recruitment and retention efforts may have been attributed to the following factors. First, careful planning and continuous monitoring throughout the recruitment process appeared to be critical for success. We set realistic recruitment targets, monitored progress, and modified our recruitment plan against those targets as needed. Recruitment was boosted when there was a decline in interest, whereas recruitment strategies were put on hold when there was a sudden increase in the expression of interest, leading to a backlog of participants requiring to be screened. A high degree of flexibility in the recruitment strategies was thus deemed necessary.

Second, our use of simple appreciation and reminder emails between assessments appeared to help with participant retention. On average, participants completed the follow-up assessment within 2 to 3 days of receiving the email link to the survey. These efforts encouraged participants to feel connected to the project, fostering an overall sense of commitment from the beginning through the completion of the study.

Third, the strength-based and delivery mode of the program may have attributed to achieving our target sample. The framing of the program as a strengthening approach to support families was important. This led to subsequent communication with potential participants in a positive way and reduced the stigma of help-seeking. For example, our formative work identified that the word “intervention” was off-putting for parents; hence, the word “program” was used in all subsequent advertising material for the trial. In addition, text messaging was a proactive approach to delivering parenting information to participants, requiring minimal effort and time commitment. The low attrition rate in our study is consistent with other studies on text messaging programs. Previous meta-analyses across a variety of text messaging–based programs found a mean retention rate of 86% [25], with retention rates ranging from 46% to 96% [26]. In our study, the ease of access is likely to have increased participation, as time constraints and logistical barriers are often raised by parents as barriers to continuation in parenting programs [27]. There has been growing acknowledgement in parenting program research for different modalities, including web-based alternatives and mHealth technology [12,28]. Our trial demonstrated the feasibility and effectiveness of providing brief preventative parenting support solely via text messages.

Finally, there was a demand for support for parents of adolescents in New Zealand. Our formative work reported that parents perceived a lack of support in the community and were interested in parenting support [18]. Many parents identified everyday parenting challenges and were interested in learning about positive parenting strategies, adolescent development, tips for improving parent-adolescent communication, and evidence-based resources. The findings were reinstated in our main trial, where parents expressed the need for more information and reported high satisfaction with the program [10].

### Limitations

Our target population comprised parents of New Zealand adolescents. Therefore, the findings may not be generalizable to studies involving other populations. Different recruitment strategies also vary substantially in cost per participant recruited, but because the study was not designed to compare the effectiveness or cost-effectiveness of recruitment strategies, we are unable to estimate the cost-effectiveness and the investment yield ratio for these strategies. Rather, our findings provide lessons to inform future studies.

Despite efforts to recruit parents from diverse population groups, our sample was predominately female, married, and of high social economic status. Many of our participants have also completed tertiary education. This is consistent with past research that reported higher levels of parent education is a predictor of parent uptake in programs [7].

Fathers were underrepresented in our study. This is a common challenge in parenting research, with fathers considered as *hard-to-reach* parents [29]. A meta-analysis of the parenting program *Triple P* found that of 4959 participants in 21 studies conducted across several countries, only 20% were fathers [30]. Underrepresentation of fathers as participants in parenting programs is concerning. Qualitative studies conducted with fathers of young children found that many fathers perceived parenting programs to be designed for mothers and that they were reluctant to seek parenting support from any formal source, as help-seeking was perceived by men as a failure and conflicted with their views on masculinity [31]. Advertising efforts that are not directed at fathers, are not related to them, or are perceived as stigmatizing are unlikely to reach fathers. Other barriers included services deemed as untrustworthy, uninterested in, or even hostile toward fathers [32,33]. Overcoming the above barriers are important to successfully engage fathers in such research. Research to understand how their engagement and participation can be maximized is urgently needed.

We also did not obtain any information from those who did not participate. Parents who do not initially engage could reveal different barriers or characteristics to those recruited into the program. Their views are therefore important and should be captured in future studies.

### Conclusions

Recruitment and retention are critical aspects of research for parenting programs, and it is unlikely that there will be a one-size-fits-all recommendation. It is therefore important that efforts are well documented to enable researchers to make more informed decisions on how and where to best recruit and therefore maximize outcome [34].

With the rapid development of technology and web-based platforms, the field would greatly benefit from empirical research designed to test the efficacy and necessity of different recruitment and retention strategies, as well as more detailed reports regarding recruitment and retention methods. Web-based recruitment strategies provide a viable means for obtaining a geographically diverse sample. Recruiting the most affected populations should be a priority, and more resources are needed to do so. Further research is needed to examine the effectiveness of tailoring recruitment strategies to different populations.

## Appendix

Multimedia Appendix 1 Facebook ad and flyer designed for recruitment.

Multimedia Appendix 2 CONSORT-eHEALTH checklist (V 1.6.1).

## Abbreviations

mHealth: mobile health
PSOC: Parenting Sense of Competence scale
