# Supplementary figures and images for: Recruitment and Retention of Parents of Adolescents in a Text Messaging Trial (MyTeen): Secondary Analysis From a Randomized Controlled Trial
Source: JMIR Pediatr Parent. 2021 Dec 20;4(4):e17723. doi: 10.2196/17723 (PMC8726057; doi:10.2196/17723)

FaceBook


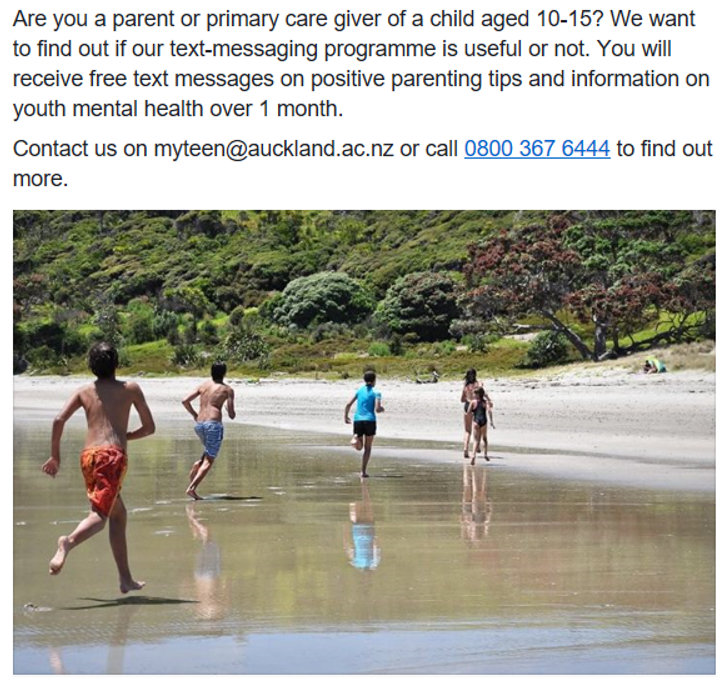


Flyer


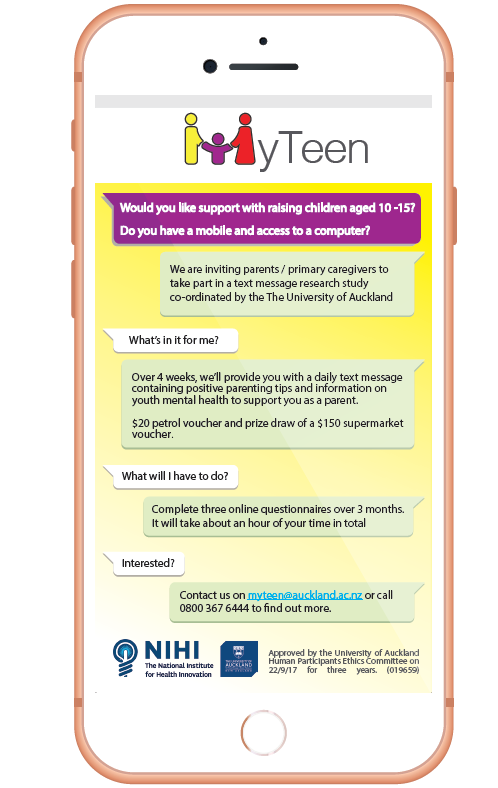

Supplement: Multimedia Appendix 1 [file pediatrics_v4i4e17723_app1.docx]
